# As Clear as Mud

**Published:** 1891-10

**Authors:** 


### AS "CLEAR AS MUD."

If "words were intended to conceal ideas" as some one has said, it is strange that educated persons should use them in such a way as to obscure or hide their meaning, when it is evident, from the nature of things, that they wish to make their meaning clear, and to be understood.

This is suggested by the singular phraseology of the 4th paragraph in the article published elsewhere in this number, in reference to a "non-resident course" proposed by the Chicago College of Physicians and Surgeons; it is certainly very ambiguous; and though one can get at the meaning from the connection, it certainly does not express it. The writer doubtless means to say that if not more than five weekly reports be made, it will be cause of complaint, but will not debar the student from credit, etc.

Without commenting on this remarkable lisense, we proceed to criticise the sentence.

The circular says:

"Not more than five weekly reports may prove unsatisfactory without debarring the student, etc."

That is to say, five weekly reports may prove unsatisfactory and may do so without debarring the student; but any number beyond that figure may not prove unsatisfactory; or if they do prove unsatisfactory, they may *not* debar the student!

Or, the first five reports must necessarily prove unsatisfactory; nevertheless, they may not debar the student. The sixth, seventh, tenth or thirtieth *cannot* prove unsatisfactory. No matter whether they be good, bad or indifferent, they will be satisfactory; or, if, perchance, any one of them should prove unsatisfactory, it does not debar the student, he gets the credit, etc., all the same.

Now we know the Faculty do not mean any such nonsense; and yet that is just what they say.

Again, in the report to the American Medical Association by the committee appointed to consider the advisability of memorializing Congress to create a Bureau (or office of Secretary) of Public health, (as published in our July number), there occurs a sentence just as ambiguous, or more so, than the above. It reads as follows:

"It will be well for the State when the medical profession is represented in the councils of the nation as weightily as can be assumed by official places and conferred dignity."

After puzzling on this till our head aches, in the vain endeavor to conceive of "the profession" being "represented as weightily as"—anything, we finally gave it up, and went to bed, positively sick from trying to understand their being—Oh, pshaw! Do official places "assume" weight? Do conferred dignities assume weight much weightier than the whole medical profession? We know nothing like this in all the literature of medicine.

